# Human Dignity and the Metaphysical Crisis in Postmodern Ethics

**DOI:** 10.1177/00243639251390484

**Published:** 2025-11-13

**Authors:** Patrícia Frantz, Francisca Rego, Stela Barbas

**Affiliations:** 1 26706 Universidade do Porto, Porto, Portugal; 2 Universidade do Sul de Santa Catarina, Palhoça, Brazil; 3 Faculdade Mar Atlântico, Rio de Janeiro, Brazil

**Keywords:** Aristotelian-Thomistic anthropology, bioethics, human dignity, nominalism, personhood, philosophy of health care and ethics

## Abstract

The concept of human dignity is frequently invoked in contemporary ethical, legal, and political discourse, yet often remains ungrounded in any coherent ontological framework. This article argues that the contemporary fragility and fragmentation of the idea of dignity stem from a deeper metaphysical crisis: the abandonment of a realist conception of human nature and the rise of nominalism. By contrasting nominalism with Aristotelian-Thomistic realism, it shows that human dignity cannot be coherently sustained without recognizing a stable and objective human essence. Through historical and philosophical analysis, the article traces how modern thought has increasingly severed the link between language and being, replacing the classical understanding of personhood with functional, procedural, or relational definitions. As a result, dignity is frequently tied to contingent attributes such as autonomy, sentience, or cognitive performance—criteria that exclude the most vulnerable members of the human family. The article examines how this metaphysical shift affects bioethical deliberations in areas such as embryonic research, end-of-life care, artificial intelligence, and transhumanism. It argues that without a stable ontological foundation, dignity becomes a rhetorical construct, vulnerable to technocratic reductionism and moral relativism. In response to contemporary objections—including pluralism, autonomy, and historicism—the article defends the ongoing relevance of a realist metaphysics. Far from suppressing freedom or diversity, it provides the necessary framework for dialogue, responsibility, and moral coherence. It concludes that defending human dignity in the postmodern age requires more than legal instruments or ethical consensus: it demands a renewed inquiry into the being of the human person, and the metaphysical foundations that make dignity not only intelligible but inviolable.

## Introduction

In recent decades, the concept of human dignity has become a central—and increasingly contested—reference point in the fields of bioethics, law, and emerging technologies. While widely invoked in policy documents, ethical frameworks, and public discourse, its philosophical foundations remain obscure, often reduced to subjective preferences, procedural consensus, or functional attributes such as autonomy or cognitive capacity. This erosion of conceptual clarity reflects not only a crisis in moral language but a deeper metaphysical rupture: the progressive abandonment of a stable ontology of the human person.

This article argues that contemporary impasses surrounding human dignity cannot be properly addressed without revisiting their metaphysical underpinnings. The contrast between *realism*, particularly in its Aristotelian-Thomistic formulation, and *nominalism*, as developed in late medieval thought and expanded in modernity, offers a decisive framework for this inquiry. Whereas realism conceives the human being as a rational and spiritual substance possessing an intrinsic essence, nominalism denies the objective reality of universals, reducing identity to linguistic or functional conventions. These differing ontologies carry profound implications for how dignity is understood, protected, or denied.

The purpose of this article is to critically compare these two metaphysical paradigms—realism and nominalism—and to evaluate their consequences for the concept of human dignity. Through a philosophical and historical approach, this study examines how each framework defines the human person, grounds moral worth, and responds to the ethical challenges posed by contemporary biopolitics and technoscientific transformations.

By recovering the metaphysical dimension of personhood, the article seeks to contribute to a more coherent and robust foundation for human dignity, one that resists both the fragmentation of postmodern relativism and the reductionism of functionalist ethics.

## The Human Dignity Discourse: Frequently Invoked, Rarely Grounded

In contemporary ethical, legal, and biotechnological discourse, the notion of human dignity is both omnipresent and conceptually unstable. As scholars have observed, the term “dignity” often functions more as a rhetorical symbol than as a philosophically grounded principle. George Kateb argues that dignity, although invoked as a universal human right, suffers from vagueness and overextension, which undermines its normative force (Kateb 2011). Similarly, Ruth Macklin provocatively claims that dignity is simply a “useless concept,” redundant in the presence of more precise notions like autonomy or respect for persons (Macklin 2003).

This ambiguity is not accidental but reflects a deeper philosophical impasse. Modernity has witnessed the progressive erosion of metaphysical conceptions of human nature, replacing them with procedural, functional, or subjective criteria. In juridical language, dignity is often tied to autonomy and rational self-determination. In bioethics, it is associated with sentience, cognitive capacity, or relational embeddedness. However, all of these frameworks raise a fundamental question: what makes every human being, regardless of condition or capacity, equally worthy of respect?

Without a stable ontological foundation—without a shared nature that precedes and grounds recognition—dignity becomes fragile and contingent. It is not enough to affirm the principle; one must ask what kind of anthropology sustains it. As Charles Taylor notes, modern pluralist societies operate with implicit “background anthropologies” that decisively shape ethical reasoning and political judgments (Taylor 1989).

The premise of this article is that recovering a coherent and enduring conception of dignity requires more than political consensus or ethical intuition. It demands a metaphysical reorientation: a renewed engagement with the being of the person as the ontological ground of moral worth. Only then can dignity cease to be a fluctuating construct and recover its status as a universal and objective principle.

## The Classical Foundations of Human Dignity

By a metaphysical framework, we refer to the investigation of the first principles of reality—those stable and universal features that make a being what it is. In the Aristotelian-Thomistic tradition, metaphysics seeks to understand the essence of things, especially the human being, not merely as a sum of biological functions or psychological states, but as a unified composite of body and soul, matter and form. Such a framework affirms that the human person has an intelligible nature—a stable “whatness”—that grounds their capacities, dignity, and purpose. Rather than constructing identity from shifting social or functional attributes, this approach roots personhood in the very act of being (*esse*), which is received from a transcendent source and ordered toward the good. To embrace a metaphysical understanding is to acknowledge that reality is not reducible to what can be measured or manipulated: it has depth, finality, and meaning that precede our constructions and judgments.

## Nominalism, Realism, and the Crisis of Human Dignity

While contemporary debates on human dignity often appear as disputes over language, policy, or methodology, they are in fact symptoms of a deeper philosophical rupture. At the heart of this rupture lies the tension between two fundamentally different views of reality: realism, which affirms the objective existence of universal natures, and nominalism, which denies them.

Nominalism, as articulated most influentially by William of Ockham^1^ in the fourteenth century, holds that only individual things exist in reality. Universal terms such as “human nature” or “personhood” do not correspond to anything real outside the mind; they are mere names (nomina) we give to similarities we perceive. In contrast, the realist tradition—upheld by Aristotle and developed by Aquinas—affirms that universals have a basis in reality and that shared natures are not just conceptual groupings, but ontological truths.

This shift from realism to nominalism fractured the link between language and being, and in doing so, it destabilized the very ground on which concepts like human dignity rest. Understanding this philosophical shift is essential to grasping how the conceptual foundations of dignity have been progressively eroded.

Within the Aristotelian-Thomistic tradition, the human being is conceived as an individual substance of rational nature, whose dignity derives from its ontological structure—that is, from the capacity to know truth, choose the good, and be ordered toward transcendence. This understanding upholds the inviolability of human life from conception to natural death, regardless of functionality, consciousness, or autonomy (Pieper 1966; Wojtyła 1979). From a Thomistic standpoint, this is grounded in the metaphysical unity of body and soul. Drawing on Aristotle's hylomorphic framework, Thomas Aquinas describes the human person as a composite of form (soul) and matter (body), where the soul is not merely a set of faculties but the substantial form—the very principle of life. Even when the body is severely impaired, as in cases of anencephaly or irreversible coma, the soul remains present so long as there is life, because it is the soul that animates the body. Human dignity, therefore, does not depend on the actualization of rational powers, but on the enduring presence of the human soul as the bearer of a natural orientation toward truth, goodness, and communion with God. This orientation, though sometimes impeded by material limitations, is never erased. Each person retains their intrinsic worth as a unique and unrepeatable creature, created in the image of God and capable in species—if not always in act—of knowing and loving Him. Thus, even the most materially diminished individual is not a defective subject, but a full member of the human family, whose dignity calls forth not pity, but reverence.

Nominalism, by contrast, denies the extra-mental reality of universals and breaks the bond between concepts and being. For William of Ockham, universals are merely names applied to multiple individuals based on perceived similarities, without any shared ontological content (Gilson 1991). This rupture between language and reality initiated a philosophical path in which the concept of “person” ceased to be an ontological datum and became defined by mutable external criteria such as cognition, autonomy, or social recognition.

This shift has profound practical implications. Without a shared ontological foundation, it becomes difficult to affirm universal dignity. The human being is increasingly interpreted through functional categories—as producer, consumer, or technological agent—and dignity becomes contingent on the capacity to act or participate in social frameworks. This perspective has shaped some strands of contemporary biocentrism and ecocentrism, in which the human species is often viewed as equal to, or even more harmful than, other forms of life (Callicott 1989; Naess 1973).

Thinkers such as Peter Singer and Paul Taylor have proposed ethical frameworks that no longer center human dignity on rational or spiritual essence, but on sentience, environmental integration, or functional performance (Singer 1979; Taylor 1986). From this viewpoint, dignity becomes conditional and negotiable, legitimizing practices such as abortion, euthanasia, embryo disposal, and even population control in the name of ecological stability (Naess 1973).

This conceptual erosion is further exacerbated by philosophical trends influenced by nominalism, such as deep ecology, where all beings are considered transient expressions of a universal energy flow without ontological hierarchy (Callicott 1989; Naess 1973). The very distinction between human and nonhuman is dissolved in favor of absolute ecological parity. Without a metaphysics that distinguishes degrees of being, ethical criteria become arbitrary.

Aristotelian-Thomistic realism, by contrast, provides a coherent ontological alternative. It acknowledges the diversity of created beings while preserving a hierarchy of forms and ends, where the human being occupies a unique place—not by arbitrary supremacy, but due to the capacity for logos and moral responsibility. It is this openness to truth and the good that founds human dignity and sustains its ethical and legal protection (Wojtyła 1979).

## Nominalism: Dissolution of Nature, Fragmentation of the Person

The metaphysical consequences of nominalism extend far beyond the denial of universals. By rejecting the extramental reality of shared natures, nominalism severs the link between being and meaning, and thus between nature and identity. What was once understood as a stable essence—such as the human being as a rational and moral agent—is now treated as a contingent construct, defined by shifting linguistic, social, or functional criteria (Gilson 1991).

In this view, language is no longer a medium for revealing the structure of reality, but a practical tool to classify phenomena according to convenience or utility. William of Ockham's insistence that universals are only mental labels imposed on individual things lays the groundwork for a world in which categories like “human,” “person,” or “dignity” are no longer rooted in ontological realities, but in sociopolitical agreements or biological performance (Gilson 1991).

This reconceptualization has profound historical and contemporary consequences. In early modernity, political theorists such as Thomas Hobbes and John Locke advanced contractualist models of society that implicitly abandoned any metaphysical grounding for the common good. The human being was reduced to a self-interested individual, defined not by an intrinsic telos, but by external relations of power and exchange. The rise of liberal individualism coincided with the growing marginalization of teleological and essentialist understandings of human nature (Pieper 1966; Wojtyła 1979).

In contemporary settings, the effects of this shift are especially visible in biotechnological discourse and in the ethics surrounding artificial intelligence. The human person is no longer considered an end in itself but a platform for optimization, enhancement, or replacement. In transhumanist literature, for example, dignity is often reinterpreted as the capacity to improve, self-program, or transcend biological limitations through technological means (Bostrom 2003). Similarly, some proponents of bioethics, such as Peter Singer, argue that moral consideration should be based not on species membership but on traits like sentience or autonomy—criteria that exclude many humans while potentially including machines or animals (Singer 1979).

This reduction of the person to cognitive or functional parameters echoes the nominalist logic: if there is no shared essence, dignity becomes a matter of performance. The notion of “person” is detached from being and redefined through ability, productivity, or utility. As a result, entire classes of human beings—such as embryos, individuals with disabilities, or the elderly—are at risk of being excluded from moral and legal protection.

Moreover, in the context of AI development, the absence of ontological criteria to distinguish human intelligence from artificial simulation aggravates this crisis. As explored by authors like Hubert Dreyfus and Joseph Weizenbaum, even the most advanced machines lack intentionality, consciousness, and moral responsibility—qualities inseparable from the metaphysical concept of personhood (Dreyfus 1992; Weizenbaum 1976). Yet, without a robust ontology, the boundary between human and nonhuman becomes blurred, opening the door to ethical relativism and technocratic control.

In summary, nominalism does not merely challenge an abstract philosophical system. It displaces the very ground on which the human being could be recognized as a bearer of inherent worth. Once the person is reduced to a node in a network of functions, rights and protections are no longer inalienable but conditional. Against this trend, only a return to a metaphysical understanding of human nature can reestablish dignity as a nonnegotiable principle.

## Contemporary Objections and Philosophical Responses

Efforts to recover a robust foundation for human dignity frequently encounter resistance within contemporary philosophical and bioethical discourse. While these objections vary in form, they tend to converge in a rejection of essentialism and of any stable account of human nature. Paradoxically, however, these critiques often underscore the very need for the kind of grounding they reject.

One common objection stems from moral pluralism. In pluralistic and secular societies, it is argued, there can be no appeal to a shared philosophical anthropology without imposing a particular worldview. Figures like Jürgen Habermas advocate for a procedural ethics based on communicative rationality, and Axel Honneth roots dignity in intersubjective recognition within social frameworks (Habermas 2003; Honneth 1996). While these models rightly emphasize the social dimension of moral life, they struggle to explain why all individuals, *prior* to recognition, ought to be respected. Without a stable human nature as reference, dignity becomes a contingent construct—always susceptible to exclusion. In contrast, a realist ontology offers not dogma but the precondition for meaningful dialogue and enduring moral commitments.

A second objection concerns autonomy. From this perspective, any appeal to a fixed human essence is seen as a threat to self-determination. Yet autonomy itself presupposes a subject that endures—capable of knowledge, choice, and responsibility. As Robert Spaemann has argued, freedom is not the invention of the self out of nothing, but a participation in the intelligibility of being (Spaemann 2006). Absent this intelligibility, autonomy loses its moral depth and collapses into arbitrariness. Far from opposing freedom, a realist account makes it possible.

Technological developments present another challenge. In an age of biotechnology and artificial intelligence, some propose that any notion of fixed human nature is obsolete. Transhumanist thinkers like Nick Bostrom envision the ethical reshaping of personhood to suit emerging capabilities (Bostrom 2003). Yet, as Hans Jonas cautioned, the very expansion of power demands a deeper moral anchor, not its abandonment (Jonas 1984). If personhood is defined by technical standards, it ceases to be a moral constant and becomes a moving target.

Historicism and language-centered philosophies add a final layer of critique. Thinkers such as Michel Foucault and Judith Butler argue that concepts like “person” and “dignity” are historically contingent and discursively constructed (Butler 2004; Foucault 1995). This view rightly exposes the political entanglements of moral language, but risks eroding the possibility of truth. Étienne Gilson reminds us that to recognize historical development is not to surrender to relativism: history presupposes a subject capable of bearing meaning across time (Gilson 1991).

Ultimately, these critiques reveal the very fragility that a realist anthropology seeks to address. Without a shared understanding of what it means to be human, “dignity” becomes a rhetorical term—appealing, but unstable. A realist account does not ignore pluralism, freedom, history, or science; rather, it offers a horizon in which these dimensions can be harmonized without dissolving the subject.

This grounding is not only philosophically coherent—it is ethically necessary. Consider a patient in a persistent vegetative state: while functionalist accounts may withdraw moral status due to the absence of consciousness or autonomy, a realist anthropology maintains that the person remains fully dignified, because their being—the unity of body and soul—endures. Likewise, an infant with anencephaly or an elderly patient with advanced dementia cannot be assessed by cognitive metrics alone. Their worth is not accidental, but intrinsic. Medical care, when rooted in this vision, is no longer guided by arbitrary standards, but by a principled recognition of the equal and unrepeatable value of every human being.

## Conclusion

Amid accelerating technological transformation and mounting ethical uncertainty, the concept of human dignity stands as both indispensable and endangered. Its widespread invocation across legal, biomedical, and political spheres is often inversely proportional to the solidity of its philosophical foundations. As this article has demonstrated, the nominalist break with classical metaphysics—most notably the denial of universals and the severing of language from being—has led to a conception of the human person that is fragmented, functional, and ultimately negotiable.

From early modern contractualism to contemporary transhumanism, from relativist ethics to the logic of artificial intelligence, the consequences of this metaphysical rupture are unmistakable: dignity becomes contingent upon cognitive performance, social recognition, or technological viability. It is reduced to a status conferred rather than an inherent quality—vulnerable to utilitarian reasoning, biotechnological redefinition, and ideological manipulation.

In contrast, the realist tradition, particularly in its Aristotelian-Thomistic articulation, offers a coherent and enduring framework. Here, the human being is a rational and spiritual substance, endowed with an intelligible nature and oriented toward truth, goodness, and transcendence. Dignity, within this vision, is not a construct but a recognition of what already is—a manifestation of the being of the person, not of social consensus or functional criteria.

To defend human dignity in this historical moment requires more than regulatory measures or moral sentiment. It demands a return to the foundational philosophical question: *what is man?* Without a metaphysical grounding of the person, dignity becomes indistinguishable from utility or preference—an empty signifier vulnerable to the pressures of ideology, technology, and cultural flux.

Reclaiming dignity thus entails more than preserving human rights; it requires restoring a vision of the human being as a bearer of intrinsic worth, whose value precedes every act, capacity, or recognition. Only such a vision can sustain a truly human bioethics—one capable of resisting the drift toward technocratic reductionism and grounding moral responsibility in the very structure of being.

## Notes

1. This reference to nominalism concerns the philosophical legacy of William of Ockham (c. 1287–1347), who denied the real existence of universal natures outside the mind. Unlike Aristotelian-Thomistic realism, which holds that concepts like *humanity* or *justice* correspond to real shared natures, Ockham argued that such terms are mere linguistic conventions applied to similar individuals. This rejection of universals did not abolish metaphysics entirely but severed the link between being and intelligibility. As a result, categories like *personhood* and *dignity* lost their ontological grounding and became contingent on shifting perceptions. Though seemingly technical, this shift laid the groundwork for modern legalism, moral voluntarism, and the current difficulty in affirming universal human dignity.
